# Clinical Lecture

**Published:** 1872-01-01

**Authors:** N. S. Davis

**Affiliations:** Professor of Clinical Medicine


### CLINICAL LECTURE

ON DISEASES OF THE SKIN—CASE OF PSORIASIS—MEDICAL WARDS OF MERCY HOSPITAL.

BY N. S. DAVIS, M. D.

*Professor of Clinical Medicine.*

Gentlemen: Before subjecting to your examination the case before us, your attention will be occupied a few moments with some comments on the general subject of cutaneous diseases. To most practitioners this is an uninviting topic, and students rarely give it that attention which its importance demands.

This class of diseases, although seldom dangerous to life, are nevertheless of frequent occurrence, many of them protracted in duration, some of them contagious or communicable, and all of them more or less annoying to the patient. Hence it is very important for every student to give them such attention, that he may be able to promptly distinguish one class from another, and to give such as may apply to him for relief the most efficient treatment. Modern writers on Dermatology or cutaneous diseases appear not to agree on any common principle of classifying the diseases in question; but some are grouped together from a supposed analogy in causation, as when they arise from *parasitic* influence; others from a common property of communicability or contagiousness; and still others from something common in the form of the eruptions. As an aid in the work of diagnosis, we think no better principle of classification has been discovered than that which was adopted many years since by Willan and Bateman, and was founded on the anatomical structure of the different varieties of cutaneous eruptions.

If we omit the modifications dependent on constitutional syphilis, and the morbid growths such as tubercle and moluscum, we may arrange all the ordinary cutaneous eruptions into five classes. The first will embrace all those in which the inflammation is so superficial as to produce only a red spot of greater or less size, without any appreciable exudation either into the cutis vera or between it and the cuticle, consequently there is neither elevation, induration nor vesication in this class, but simple red spots. These spots may be very small and generally diffused over the surface, as in scarlatina, or they may be small and grouped in clusters, as in measles, or they may be larger and more isolated, as in roseola and erythema. These form the *Exanthematous* class. The second will embrace all those eruptions in which the inflammation is sufficient to cause a serous exudation between the cutis vera and the cuticle, simply elevating the latter into the form of a vesicle filled with lymph or serum, but without any plastic exudation sufficient to give hardness or thickening of the cutis vera. The vesicles may be very small as in scabies and eczema, or larger as in varicella, herpes and pemphigus. These are called vesiculæ, or the vesicular class.

The third will embrace those in which the inflammation is sufficiently intense not only to cause an exudation of lymph or serum between the cutis vera and cuticle, forming a vesicle, but in addition, sufficient plastic exudation into the true skin and the subjacent tissue to furnish a more or less elevated and indurated base on which the vesicle will rest. Suppuration or the formation of pus in the vesicle during some stage of its progress, is also a constant occurrence in all the varieties included in this group. Hence they are called pustulæ or the pustular class. The more important examples of this class are Ecthyma, Impetigo, Mentagra, Porrigo, and the pustules of Variola and Vaccina.

The fourth embraces those eruptions in which the inflammation causes a minute amount of plastic exudation into the cutis vera, causing a slight induration and elevation, but without either vesication or suppuration. The small indurated and elevated spots thus formed are called papules; hence the group are called papulæ or the papular class. The chief varieties of disease included in this class are Lichen, Strofulus and Porrigo.

The fifth and last class embraces those affections of the skin characterized by such a chronic grade of inflammation as causes spots of variable size, in which the cutis vera is more or less thickened, and from which the cuticle is constantly being exfoliated in the form of laminæ or dry scales. This latter circumstance has caused this group to be called squamæ or the squamous class. The varieties of disease properly belonging to this class are Ptyriasis, Lepra, Psoriasis, and perhaps the leprosy of the ancients. Icthyosis or fish-skin, which has sometimes been included in this class, is generally a congenital defect rather than a cutaneous inflammation.

With these general remarks you will be prepared to proceed with the examination of the case before you. The patient is a young man of foreign birth, a sailor by occupation, and apparently in fair general health, but presents spots of cutaneous disease on his legs, thighs, arms, and a few on the trunk of the body. The first step in the matter of diagnosis is to determine to which of the classes we have named the case belongs. As you individually proceed to examine carefully those spots on the uncovered parts of the patient's legs you will readily see that they are not made up of minute red points like the rash of measles or scarlet fever, and can not therefore belong to the exanthematous class. Neither can you find any vesicles either filled with lymph or broken and weeping a serous fluid, as in the vesiculæ; nor any pustules filled with pus and standing on a hard base as in the pustular class. You look equally in vain for the small hard elevations or papules which characterize the fourth or papular class.

What, then, have you in this case? Simply scattered spots, varying in size from the circumference of a pea to that of a silver half dollar, of irregular outline, deep red color, slightly elevated and rough, and on the surface of which appear dry, white, thin laminæ of exfoliating cuticle. These characteristics place it directly in the class of squamæ or scaly diseases. Having designated the class to which it belongs, the next step is to ascertain which variety of that class it represents. There being but three varieties of this class met with in this country, the identification is easy. The Ptyriasis seldom if ever exists on any part of the skin except that covered with hair, as the scalp, arm-pits and pubes. It is accompanied by only slight redness, and the exfoliating cuticle is in the form of very small scales, like dandruff. None of these circumstances apply to the case before you. Lepra may come on any part of the surface in red, dry, rough spots, at first the size of a half dime. They spread regularly on the circumference and heal in the centre, and thereby soon assume a circular or ring form; hence the popular name of "ring-worm." The spots before you have no such regular ring form, although some of them have existed and been slowly increasing for many months. The only remaining variety of the squamous class is Psoriasis, of which the spots on the legs before you are perfect examples. You see them variable in size, irregular in outline, dry, rough, deep red, with large thin white laminæ of cuticle on the surface. The disease is chronic, the spots generally increasing in size slowly, and continuing for months and years unless interfered with by remedies. Sometimes the spots come in the palms of the hands and present a very hard, rough, and sometimes fissured appearance. It is then called Psoriasis Palmaris or "baker's itch." The disease does not appear to be dependent on any particular constitutional derangement, though often connected with constitutional syphilis. In the latter case the spots have a more livid or coppery color. Except in the latter class of cases, the most important part of the treatment consists in proper local applications. If there is any manifest derangement of the digestive or other important functions, it should be corrected. In the absence of any special indications, it may be advantageous to give small doses of some one of the arsenical preparations. We shall give this man eight drops of Donovan's Solution three times a day for the first ten days, and have the spots rubbed thoroughly night and morning with an ointment of iodide of sulphur 20 grs. to the oz. of cerate. After the first ten days we will substitute an ointment of ammoniated mercury, 20 grs.; pulv. g. camph. 8 grs.; tinct. bloodroot fl. ʒ ss., rubbed together and mixed with simple cerate ℥ j. Let this be applied every night and the surface wet with glycerine in the morning.

A full alkaline bath should be used twice a week; the diet plain, exercise in the open air moderate, and all stimulants avoided.

